# Redefine Hyperprogressive Disease During Treatment With Immune-Checkpoint Inhibitors in Patients With Gastrointestinal Cancer

**DOI:** 10.3389/fonc.2021.761110

**Published:** 2021-11-09

**Authors:** Zhenghang Wang, Chang Liu, Yuezong Bai, Xiaochen Zhao, Longgang Cui, Zhi Peng, Xiaotian Zhang, Xicheng Wang, Zhengyi Zhao, Jian Li, Lin Shen

**Affiliations:** ^1^ Department of Gastrointestinal Oncology, Key Laboratory of Carcinogenesis and Translational Research (Ministry of Education), Peking University Cancer Hospital & Institute, Beijing, China; ^2^ Medical Affairs, 3D Medicines Inc., Shanghai, China

**Keywords:** hyperprogressive disease, gastrointestinal cancer, immune checkpoint inhibitors, circulating tumor DNA, next-generation sequencing

## Abstract

**Objective:**

Emerging evidence showed that immune checkpoint inhibitors (ICIs) lead to hyperprogressive disease (HPD) in a small proportion of patients. There is no well-recognized standard for the evaluation of HPD. Comprehensive exploration of HPD definition system in gastrointestinal cancer treated with ICI is lacking to date.

**Methods:**

A total of 126 patients with advanced or metastatic gastrointestinal cancer treated with ICI monotherapy were analyzed. Seven definitions of HPD were defined with tumor growth kinetics (TGK) or tumor growth rate (TGR) by including new lesions or not, and with different cutoffs. Incidence and performance of different criteria were compared. Clinicopathologic characteristics and baseline genomic variations associated with HPD were also explored.

**Results:**

Tumor growth kinetics ratio of more than two fold that incorporated new lesions into calculation of HPD outperformed other definitions by successfully stratifying 14 patients (11.1%) with both accelerated disease progression (median PFS, 1.62 *versus* 1.93 months; hazard ratio, 1.85; 95% CI, 0.98 to 3.48; P = 0.059) and worse overall survival (median OS, 3.97 *versus* 10.23 months; hazard ratio, 2.30; 95% CI, 1.11 to 4.78; P = 0.021). Baseline genomic alterations in circulating tumor DNA, including SMARCA2, MSH6, APC signaling pathway, and Wnt signaling pathway, might be associated with the risk of HPD.

**Conclusion:**

Incorporating new lesions emerging during the treatment was shown to be reliable for the assessment of TGK. TGK serves as a more convenient way to reflect tumor growth acceleration compared with TGR. Genomic alterations were suggested to be associated with the occurrence of HPD.

## Introduction

Cancer treatment has entered the era of immunotherapy. Immunotherapy with immune checkpoint inhibitors (ICIs) has made great progress in gastrointestinal (GI) cancer treatment. Currently, FDA-approved indications for GI cancer include pembrolizumab monotherapy, nivolumab as monotherapy, or in combination with ipilimumab for microsatellite instability-high (MSI-H) or mismatch repair deficient (dMMR) colorectal (1–3); pembrolizumab for metastatic or advanced gastric and esophageal cancers with PD-L1 positive tumors (4); nivolumab for advanced gastric or gastroesophageal junction cancer refractory to or intolerant of at least two previous chemotherapy regimens. In the phase 3, ATTRACTION-2 study, nivolumab showed superior survival benefits over placebo in Asian patients with heavily pretreated advanced gastric or gastroesophageal junction cancer (5). REGONIVO study showed encouraging efficacy of nivolumab plus regorafenib in microsatellite stable (MSS) colorectal or gastric cancer patients (6).

However, emerging evidences showed that ICI treatment can sometimes lead to hyperprogressive disease (HPD), a paradoxical boost in tumor growth. HPD was initially reported and defined by Champiat S. et al. in 2017 (7). Thereafter, the occurrence of HPD during immunotherapy has been reported in many tumor types, including non-small-cell lung cancer (NSCLC), melanoma, head and neck squamous cell carcinoma, gastric cancer, and hepatocellular cancer (7–11). Various criteria have been developed to define HPD to capture the rapid tumor growth in this specific scenario. HPD was defined by Champiat S. et al. as tumor progression according to Response Evaluation Criteria in Solid Tumors version 1.1 (RECIST v1.1) at the first evaluation and a two fold or greater increase in tumor growth rate (TGR) during ICI therapy in comparison with pretreatment kinetics (7). Ferrara R. et al. defined HPD as disease progression at first evaluation with an increase of TGR exceeding 50%, which was validated in NSCLC patients (12). Notably, disease progression of some patients is driven by new metastases, which were excluded from the calculation of TGR in the previous reports (7, 11, 12). We previously reported evaluation of HPD with the diameters of measurable new lesions taken into account in the total tumor burden in tumors of digestive system treated with immunotherapy, and HPD was defined as tumor growth kinetics (TGK) ratio ≥ 2 (13).

HPD leads to accelerated disease deterioration and shortened survival; therefore, identifying patients with HPD is critical for adjusting treatment strategy. However, there is no consensus regarding the evaluation of HPD status. In addition, tumor biological behavior and genetic characteristics vary in different type of tumors, thus leading to diverse response and progression pattern upon immunotherapy. Systematic exploration of HPD definition in gastrointestinal cancer is lacking.

Furthermore, the mechanism underlying the occurrence of HPD still remained unclear. *MDM2* amplification, *epidermal growth factor receptor (EGFR)* mutations, and antibody-Fc/FcR interaction on macrophages were the potential mechanisms that were reported to be potentially associated with HPD by far (14, 15).

In the present study, we aimed to develop an optimized criterion for HPD evaluation in gastrointestinal cancer patients treated with PD-1/L1 inhibitors through rational design and validated its prognostic value through comparison with other mainstream HPD definitions, with additional exploration of potential predictors of HPD in gastrointestinal cancer patients.

## Materials and Methods

### Patients

Clinicopathological information and treatment outcomes of patients with gastrointestinal adenocarcinoma who received PD-1/PD-L1 inhibitors as monotherapy from February 2016 to January 2020 in Beijing Cancer Hospital were reviewed and retrospectively collected. The pathological and imaging results of all cases were retrospectively reviewed by two pathologists and two radiologists, respectively. Biochemical profiling was conducted before and during the immunotherapy. Next-generation sequencing (NGS) was applied on blood-derived circulating tumor DNA (ctDNA) using a 150-gene panel at 3DMed Clinical Laboratory Inc., a College of American Pathologists (CAP)–accredited and Clinical Laboratory Improvement Amendments (CLIA)–certified laboratory of 3D Medicines Inc. Blood samples were obtained from each patient within 7 days before ICI treatment.

### Definitions of HPD

CT scans were conducted within 6 weeks before immunotherapy, within 7 days before treatment initiation, and at least 2 weeks after the immunotherapy. The interval of CT scans was at least 2 weeks. HPD was defined according to volume changes or diameter changes. TGR and TGK were applied to express volume changes and diameter changes, respectively. For TGR calculation, S is the sum of the diameters of target lesions with or without new measurable lesions emerging between the two CT scans. Tumor volume (V) was presented as V = 4πR^3^/3, where R is equal to S/2. Vt, the tumor volume at time t, expressed in month, is equal to Vt = V0*exp(TG*t), where V0 is the volume at baseline, and TG is the growth rate. TG equals to TG=3*Log(Dt/D0)/t. TGR is the percentage increase of tumor volume per month, which is calculated using the following formula: TGR = 100 [exp(TG) -1] (16). TGK was expressed as changes of S (the same as TGR) per month (8).

One major feature of HPD is its more aggressive behavior and worse survival outcomes compared to non-HPD progressive disease, which is the key criterion to evaluate the reliability of HPD definitions. The definition of HPD varies in the previous reports (7, 12, 13) with the major differences in three aspects: (1) the calculation method of tumor growth pattern; (2) the inclusion or exclusion of new lesions; (3) the threshold of tumor growth speed during ICI treatment.

In light of all the factors mentioned above, seven different definition criteria of HPD were established, some of which were reported previously (7, 8, 12, 17). Details of the calculation and threshold of every definition are described in **Table 1**.

**Table 1:** Definitions of hyperprogressive disease.

Definitions of HPD	Calculation of tumor growth pattern	New lesions	Criteria of HPD
Definition 1	TGpre=3 Log(Sbaseline/Spre)/t. TGRpre = 100 (exp(TG) -1). TGpost=3 Log(Spost/Sbaseline)/t. TGRpost = 100 (exp(TG) -1).	Included	(TGRpost-TGRpre)>50%
Definition 2	TGpre=3 Log(Sbaseline/Spre)/t. TGRpre = 100 (exp(TG) -1). TGpost=3 Log(Spost/Sbaseline)/t. TGRpost = 100 (exp(TG) -1).	Included	TGRpost/TGRpre>2
Definition 3	TGKpre=(Sbaseline-Spre)/(Tbaseline-Tpre). TGKpost=(Spost-Sbaseline)/(Tpost-Tbaseline).	Included	TGKpost/TGKpre>2
Definition 4	TGpre=3 Log(Sbaseline/Spre)/t. TGRpre = 100 (exp(TG) -1). TGpost=3 Log(Spost/Sbaseline)/t. TGRpost = 100 (exp(TG) -1).	Not included	(TGRpost-TGRpre)>50%
Definition 5	TGpre=3 Log(Sbaseline/Spre)/t. TGRpre = 100 (exp(TG) -1). TGpost=3 Log(Spost/Sbaseline)/t. TGRpost = 100 (exp(TG) -1).	Not included	TGRpost/TGRpre>2
Definition 6	TGKpre=(Sbaseline-Spre)/(Tbaseline-Tpre). TGKpost=(Spost-Sbaseline)/(Tpost-Tbaseline).	Not included	TGKpost/TGKpre>2
Definition 7	RECIST 1.1	Included	1.4 * baseline sum target lesions or 1.2 * baseline sum target lesions + new lesions in at least two different organs

S, sum of the diameters of target lesions with/without new lesions ermerging during treatment; TG, tumor growth; TGK, tumor growth kinetics; TGR, tumor growth rate.

A switch of treatment regimen and intense supportive care should always be considered after HPD owing to its high mortality. Hence, a reliable definition of HPD should meet at least the following two requirements: (1) the identified HPD cases should have significantly shorter OS than those with non-HPD progression disease; and (2) this definition could identify as many as possible cases with poorer OS as HPD. In addition, the ease of calculation is also a factor that should be taken into consideration, if this algorithm is to be adopted in daily clinical care.

### Molecular Testing

The ctDNA extraction, library preparation, capture sequencing, and variants calling have been described previously (18). The captured DNAs were loaded into NextSeq 500 (Illumina) for 75 bp paired-end sequencing according to the manufacturer’s instructions. Somatic and germline alternations were identified, and the clinicopathological information was collected. Blood-based tumor mutational burden (bTMB) was calculated as the sum of somatic single nucleotide variants and insertion-deletions in examined coding region. Tissue MSI status and MMR protein expression were respectively confirmed by PCR and immunohistochemistry test when sufficient paired tumor tissues were available. This study was approved by the ethics committees of Beijing Cancer Hospital, and all patients provided written informed consent. Patient identity protection was maintained throughout the study.

### Assessment of Clinical Outcomes

Tumor burden at pre-baseline, baseline, and post-baseline were evaluated in all patients with radiological reports according to RECIST v1.1. Overall survival (OS) was defined as the time from the start of anti-PD-1/L1 treatment until death due to any cause. Progression-free survival (PFS) was defined as the time from the start of anti-PD-1/L1 treatment until disease progression or death.Differences between two groups were assessed by Student’s t test for normally distributed variables or by the Mann-Whitney U-test for non-normal distributed ones. Chi-square test or Fisher’s exact test was used to examine the difference of categorical variables between two groups. For OS analysis, Kaplan-Meier curves were compared by using log-rank test, and the hazard ratio (HR) was determined through a Cox regression model. All reported *P* values were two-tailed, and *P* <.05 was considered statistically significant. Data were analyzed using GraphPad Prism (version 8.02, GraphPad Software, USA), SPSS statistical software (version 20.0, SPSS, IBM Corporation, USA), and R version 3.5.0 software (www.r-project.org).

## Results

### Patients Characteristics

**Figure 1:**
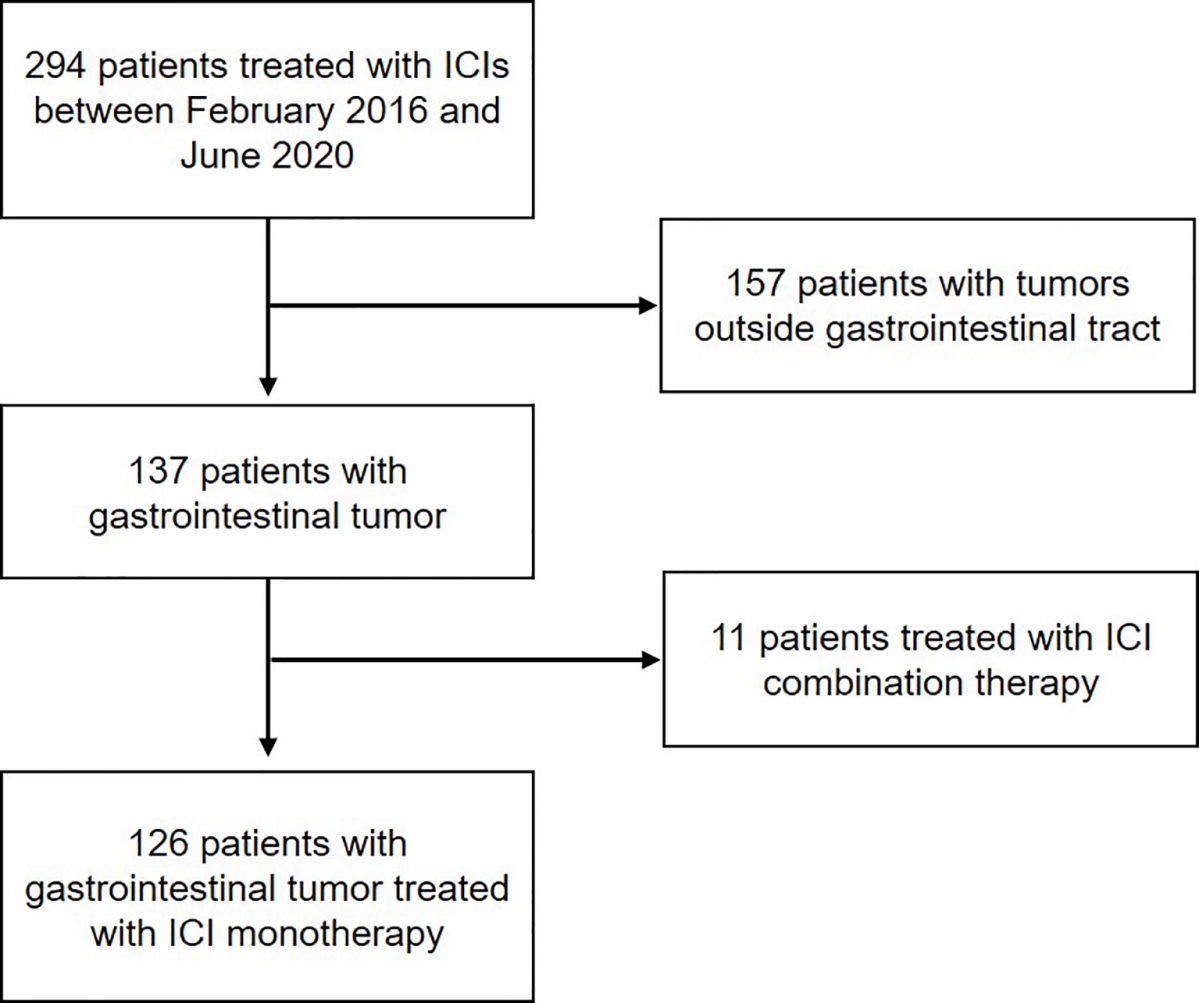
Workflow of the study. Flow diagram illustrating the patients included for the analytical process.

A total of 294 consecutive patients with advanced or metastatic cancer treated with anti-PD-1/L1 therapy from February 2016 to June 2020 in our center were retrospectively screened. Amongst all, 168 patients were excluded due to diagnosis of tumor types other than gastrointestinal adenocarcinoma or receiving ICI combination therapy. In all, 126 patients were included for our analysis, including 83 patients treated with anti-PD-1 and 43 patients treated with anti-PD-L1 (**Figure 1**).

The demographic and clinicopathologic characteristics of the 126 patients are described in **Table 2**. The median age was 57.5 (range, 44–66), and 65.1% (82 of 126) of the patients were male. Overall, 59 patients were diagnosed with gastric cancer and 67 patients with intestinal cancer, including 59 colorectal cancer, 4 duodenal cancer, 3 small bowl cancer, and 1 appendix cancer. Seventy-one (56.3%) patients were identified as MSI-H, including 19 gastric cancer and 52 intestinal cancer patients.

**Table 2:** Baseline characteristics.

Characteristics (n=126)	No. of patients (%)
Age, median (IQR range)	57.5 (44–66)
Sex, n (%)	
Male	82 (65.1)
Female	44 (34.9)
Tumor type, n (%)	
Stomach	59 (46.8)
Duodenum	4 (3.2)
Small intestine	3 (2.4)
Appendix	1 (0.8)
Colorectal	59 (46.8)
Prior lines of treatment, n(%)	
0	13 (10.3)
1	47 (37.3)
2	46 (36.5)
>=3	20 (15.9)
Immunotherapy type, n (%)	
Anti-PD-L1	43 (34.1)
Anti-PD-1	83 (65.9)
ECOG performance status, n (%)	
0	39 (31.0)
1	87 (69.0)
Organs with metastases, n (%)	
<3	59 (46.8)
≥3	31 (24.6)
Liver metastasis, n (%)	31 (24.6)
Peritoneal metastasis, n (%)	29 (23.0)
Lung metastasis, n (%)	20 (15.9)
Lymph node metastasis, n (%)	57 (45.2)
HBV, n (%)	39 (40.0)
dMMR/MSI-H, n (%)	71 (56.3)

PD-L1, programmed cell death ligand 1; PD-1, programmed cell death 1; ECOG performance status, Eastern Cooperative Oncology Group performance status; HBV, hepatitis B virus; dMMR, mismatch repair deficient; MSI-H, microsatellite instability-high.

The median follow-up was 10.50 months (95% CI, 8.30–13.82 months). Response rate was 31.0% (39 of 126), 30.5% (18 of 59), and 31.3% (16 of 57) in the total population, the gastric cancer, and the intestinal cancer patients, respectively. The median OS was 19.20 months (95% CI, 15.17–23.22 months) in the overall cohort, 11.37 months (95% CI, 5.97–16.77) for patients with gastric cancer, and not reached for patients with intestinal cancer. The median PFS was 5.70 months (95% CI, 3.18–8.23 months), 4.20 months (95% CI, 1.47–6.93 months), and 4.21 months (95% CI, 0–15.15 months) in overall, gastric cancer, and intestinal cancer cohort, respectively.

### Comparison of the Incidence and Performance of Different HPD Definitions

Fifty-one patients experienced progressive disease at first radiological evaluation during ICI treatment, including 25 gastric cancer, 22 colorectal cancer, 2 small intestinal cancer, 1 appendix cancer, and 1 duodenal cancer. No pseudoprogression, progressive disease followed by tumor regression, was observed in our cohort. Median interval of radiological evaluation from pre-baseline to baseline was 43 days (range, 14–181 days); from baseline to post-baseline was 64 days (range, 25–126 days).

**Figure 2:**
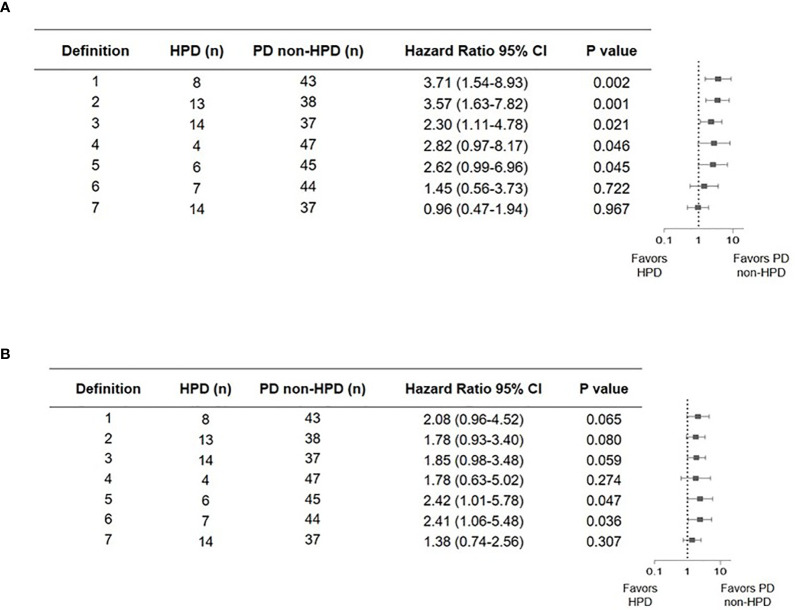
Forest plot of hazard ratios from comparison of overall survival **(A)** and progression-free survival **(B)** in patients who experienced hyperprogressive disease versus those who experienced progressive but not hyperprogressive disease with seven definitions. Squares represent hazard ratio (HR). Horizontal lines indicate the 95% CIs. HPD, hyperprogressive disease; CI, confidence interval.

Direct comparisons on incidence and performance were carried out between different HPD definitions among 51 patients with progressive disease. Clinical characteristics and survival outcome of patients with HPD identified with different definitions are listed in **Table 3** and **Figure 2**. Compared to definition 4 to 6, in which new lesions emerging during ICI therapy were not counted as tumor growth, definitions 1, 2, 3, and 7 identified more patients with HPD, indicating that excluding new lesions might underestimate the incidence of HPD, which is readily comprehensible as a proportion of patients have disease progression due to the appearance of new lesions. Definitions 1 to 3 identified patients with significantly worse OS compared with non-HPD progressive disease, while definition 3 distinguished maximum number of patients with tumor growth acceleration. Although definition 7 identified 14 patients from overall cohort, it failed to distinguish patients with accelerated tumor progression, as median OS of HPD and non-HPD progressive disease were 7.43 *versus* 8.87 months, respectively (hazard ratio, 0.96; 95% CI, 0.47 to 1.94; P = 0.97).

**Table 3:** Characteristics of patients defined as HPD according to different definitions.

Component	Definition 1	Definition 2	Definition 3	Definition 4	Definition 5	Definition 6	Definition 7
Incidence of HPD, n (% in overall cohort)	8 (6.3)	13 (10.3)	14 (11.1)	4 (3.2)	6 (4.8)	7 (5.6)	14 (11.1)
Cancer types defined as HPD, n (% in HPD)							
Stomach	4 (50.0)	6 (46.2)	7 (50.0)	2 (50.0)	2 (33.3)	3 (42.9)	5 (35.7)
Duodenum	0	1 (7.7)	1 (7.1)		1 (16.7)	1 (14.3)	0
Small intestine	1 (12.5)	1 (7.7)	1 (7.1)	1 (25.0)	1 (16.7)	1 (14.3)	2 (14.3)
Appendix	0	0	0	0	0	0	0
Colorectal	3 (37.5)	5 (38.5)	5 (35.7)	1 (25.0)	2 (33.3)	2 (28.6)	7 (50.0)
MMR/MSI status, n(% in HPD)							
dMMR/MSI-H	3 (37.5)	5 (38.5)	4 (28.6)	1 (25.0)	2 (33.3)	2 (28.6)	5 (35.7)
pMMR/MSS	4 (50.0)	7 (53.8)	9 (64.3)	3 (75.0)	4 (66.7)	5 (71.4)	8 (57.1)
NA	1 (12.5)	1 (7.7)	1 (7.1)	0	0	0	1 (7.1)
Prior lines of treatment, n(% in HPD)							
0	0	0	0	0	0	0	0
1	3 (37.5)	3 (23.1)	4 (28.6)	1 (25.0)	1 (16.7)	2 (28.6)	2 (14.3)
2	3 (37.5)	7 (53.8)	7 (50.0)	1 (25.0)	3 (50.0)	3 (42.9)	3 (42.9)
>=3	2 (25.0)	3 (23.1)	3 (21.4)	2 (50.0)	2 (33.3)	2 (28.6)	3 (42.9)
Hazard ratio (95% CI) for OS between HPD *vs.* non-HPD progressive disease	3.71 (1.54–8.93)	3.57 (1.63–7.82)	2.30 (1.11–4.78)	2.82 (0.97–8.17)	2.62 (0.99–6.96)	1.45 (0.56–3.73)	0.96 (4.07–1.94)
P value for OS comparison between HPD *vs.* non-HPD progressive disease	0.002	0.001	0.021	0.046	0.045	0.722	0.967

dMMR, mismatch repair deficient; MSI-H, microsatellite instability-high; pMMR, mismatch repair proficient; MSS, microsatellite stability; OS, overall survival.

Taken together, definition 3 outperformed other criteria by successfully stratifying patients with both more aggressive disease progression and worse survival outcome. Higher number of patients with HPD was screened out by definition 3. Furthermore, calculation of tumor growth kinetics is also readily accessible, which allows widespread application in clinical practice.

### Clinical Characteristics and Survival Outcome of HPD Subgroup According to Definition 3

**Figure 3:**
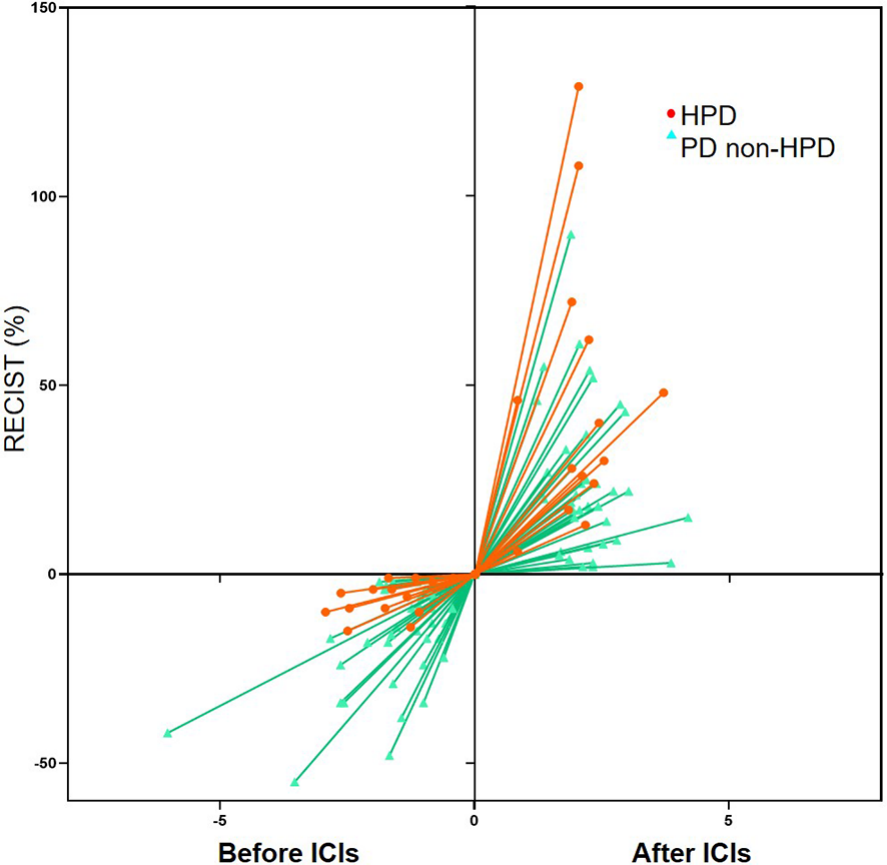
Spider plot depicting the percent change in the sum of the longest diameters of target lesions and new lesions (RECIST) before immune checkpoint inhibitors (ICIs) and after ICIs periods in the 51 evaluable patients according to definition 3. HPD, hyperprogressive disease; PD, progressive disease.

**Figure 4:**
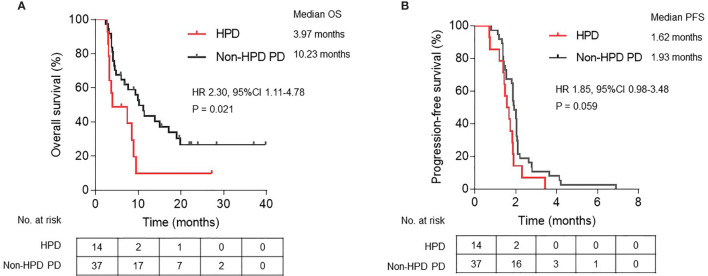
Kaplan-Meier plots of **(A)** overall survival (OS) and **(B)** progression-free survival (PFS) in patients defined as HPD compared with non-HPD progressive disease. HPD, hyperprogressive disease; PD, progressive disease; HR, hazard ratio; CI, confidence interval.

Fourteen patients were defined as HPD by definition 3, including seven gastric cancer, six colorectal cancer, and one duodenal cancer. TGKpre ranged from 0.59 to 11.20 and TGKpost ranged from 2.14 to 47.19 in HPD subgroup. Spider plot was used to depict the percent change in the sum of the longest diameters of target lesions and new lesions before and after ICI treatment in the 51 evaluable patients according to definition 3 (**Figure 3** **)**. Significantly shortened OS was observed in patients who met HPD criteria of definition 3 compared with patients with non-HPD progressive disease (median OS, 3.97 *versus* 10.23 months; hazard ratio, 2.30; 95% CI, 1.11 to 4.78; P = 0.021) (**Figure 4A** **)**. Furthermore, median PFS in patients with HPD was 1.62 months, which also tended to be worse than that of non-HPD progressive disease with median PFS of 1.93 months (hazard ratio, 1.85; 95% CI, 0.98 to 3.48; P = 0.059) (**Figure 4B** **)**. Although the PFS evaluation could be influenced by the interval of image scan, survival analysis still indicated that HPD during immunotherapy is associated not only with shorter survival but also with more aggressive disease, which could explain the poor clinical outcomes of this subgroup. Survival curves based on other six definitions are also displayed in **Supplementary Figure 1**.

Seven patients would be underestimated as non-HPD progressive disease if new lesions emerging during ICI treatment are not calculated into tumor growth. This discordant subgroup of patients still had significantly worse OS comparing with non-HPD progressive disease (hazard ratio, 2.98; 95% CI 1.05 to 8.46; P = 0.004) **(** **Supplementary Figure 2** **)**. Within 14 patients defined as HPD by definition 3, discordant subgroup had even numerical worse OS compared with the rest seven patients, although the OS difference was not statistically significant (median OS, 3.97 *versus* 7.43 months; hazard ratio, 1.64; 95% CI, 0.46 to 5.88; P = 0.444) (**Supplementary Figure 2**).

### Clinical Factors and Genomic Alterations Associated With HPD

The potential factors associated with HPD were further analyzed in our cohort **(** **Table 4** **)**. Baseline clinicopathological characteristics, blood biochemical indexes, and genomic alterations were compared between patients with HPD and non-HPD progressive disease. No association was observed between the occurrence of HPD with patients’ age, gender, primary tumor site, lines of treatment, treatment regimen, and MSI status. Analysis of baseline blood biochemical indexes and peripheral blood cell counts showed no significant difference between HPD and non-HPD progressive disease.

**Table 4:** Clinical factors and genomic alterations associated with HPD.

Characteristics	HPD (n=14)	non-HPD PD (n=37)	P value
Age			0.715
≥65, n (%)	4 (28.6)	8 (21.6)	
<65, n (%)	10 (71.4)	29 (78.4)	
Male, n (%)	7 (50)	25 (67.6)	0.334
ECOG			0.301
0	6 (42.9)	9 (24.3)	
1–2	8 (57.1)	28 (75.7)	
Primary tumor site			>0.999
Gastric cancer	7 (50)	18 (48.6)	
Intestinal cancer	7 (50)	19 (51.4)	
Treatment lines, n (%)			>0.999
<3	4 (28.6)	12 (32.4)	
≥3	10 (71.4)	25 (67.6)	
Treatment, n (%)			0.749
Anti-PD-L1	4 (28.6)	13 (35.1)	
Anti-PD-1	10 (71.4)	24 (64.9)	
MMR/MSI status			0.743
pMMR/MSS	9 (64.3)	21 (56.8)	
dMMR/MSI-H	4 (28.6)	13 (35.1)	
NA	1 (7.1)	3 (8.1)	
Elevated baseline CA 19-9	9 (64.3)	18 (48.6)	0.363
Elevated baseline CEA	10 (71.4)	22 (59.5)	0.527
Elevated baseline LDH	9 (64.3)	18 (48.6)	0.363
Baseline hemoglobin <120 g/L	4 (28.6)	15 (40.5)	0.527
Baseline albumin <35 g/L	1 (7.1)	1 (2.7)	0.478
Baseline NLR			0.198
NLR<Median (3.14)	11 (78.6)	20 (54.1)	
NLR≥Median (3.14)	3 (21.4)	17 (45.9)	
ΔNLR			>0.999
ΔNLR ≤ 0	3 (21.4)	9 (24.3)	
ΔNLR>0	11 (78.6)	27 (73)	
Baseline PLR			0.202
PLR<Median (171.68)	11 (78.6)	21 (56.8)	
PLR≥Median (171.68)	3 (21.4)	16 (43.2)	
ΔPLR			
ΔPLR ≤ 0	4 (28.6)	10 (27.0)	>0.999
ΔPLR>0	10 (71.4)	26 (70.3)	
Ts/TC			>0.999
Ts/TC<median (26.8)	2 (14.3)	10 (27.0)	
Ts/TC≥median (26.8)	4 (28.6)	15 (40.5)	
Genomic alteration associated with HPD, n (% in patients sequenced)			
MSH6	0	10(38.5)	**0.039**
SMARCA2	3 (33.3)	1 (3.8)	**0.041**
Wnt pathway	2 (22.2)	16 (61.5)	0.060
APC pathway	2 (22.2)	18 (69.2)	**0.021**

ECOG performance status, Eastern Cooperative Oncology Group performance status; dMMR, mismatch repair deficient; MSI-H, microsatellite instability-high; pMMR, mismatch repair proficient; MSS, microsatellite stability; NLR, neutrophil-to-lymphocyte ratio; ΔNLR, post-treatment NLR minus pre-treatment NLR; PLR, platelet-to-lymphocyte ratio; ΔPLR, post-treatment PLR minus pre-treatment PLR; Ts/TC, CD3+CD8+ T cell. Statistically significant values are indicated in bold.

Within 51 patients with progressive disease, ctDNA derived from blood samples prior to immunotherapy were collected from 35 patients and sequenced *via* target NGS analysis, including nine patients diagnosed as HPD (**Supplementary Figure 3** and **Supplementary Table 2** **)**. Firstly, we compared the incidence of every single gene between HPD and non-HPD subgroups. Patients with *MSH6* mutation were found to have a lower incidence of HPD (Fisher exact test P = 0.039). On the contrary, patients with *SMARCA2* mutation had higher incidence of HPD (Fisher exact test P = 0.041). Furthermore, we also analyzed if genomic alterations in signaling pathway would be related to HPD. Patients with alterations in Wnt signaling pathway, including *AMER1*, *APC*, *AXIN1*, *AXIN2*, *CHD4*, *CTNNB1*, *GSK3B*, *LEF1*, *LZTR1*, *RNF43*, *TCF7L2*, *WIF1*, and *ZNRF3*, were observed to have borderline lower incidence of HPD (Fisher exact test P = 0.059), while patients with alterations in APC signaling pathway, covering *AMER1*, *APC*, *AXIN1*, *CDH1*, *CTNNB1*, *HNF1A*, *NF2*, *RNF43*, and *SOX9*, had higher incidence of HPD (Fisher exact test P = 0.021). From the results above, genomic variates in SMARCA2 gene or APC signaling pathway might be associated with the higher risk of HPD. On the other hand, patients with *MSH6* gene or Wnt signaling pathway alterations might have lower risk of HPD. The bTMB was also analyzed, and no significant difference was found between HPD and non-HPD progressive disease subgroups (P = 0.316).

## Discussion

In this study, we have for the first time conducted a comprehensive comparative analysis into the methods to define HPD in gastrointestinal cancer. We have provided a definition of HPD, which outperformed other six different criteria systems and could serve as reliable criteria for capturing the disease hyperprogression status of cancer patients and distinguish it from regular disease progression (non-HPD progressive disease). Analysis regarding genomic mutations in ctDNA from patients with progressive disease found that several genes and signaling pathways might be associated with HPD.

The concept of HPD has been reported in several previous studies; however, there is no consensus on the definition of HPD status to date. A reliable definition of HPD should be able to identify patients with shorter survival as HPD, e.g., identified HPD cases should have significantly poorer OS than non-HPD. Criteria system in several early studies assessed tumor growth only with the target lesions while new lesions appearing during treatment were excluded (7, 10, 12, 19). A proportion of cancer patients presenting rapid disease progression were attributed to new lesions and metastases (20); thus, the exclusion of new lesions in tumor burden evaluation would underestimate the tumor growth kinetics and may lead to the misdiagnosis in HPD. One recent study has compared two definitions for HPD calculation in patients with solid tumors (17). The authors proposed that disease hyperprogression assessed by the occurrence of early progressive disease plus the increase of measurable lesions and/or appearance of new lesions is superior to the criteria for tumor growth rate measurement in HPD determination, which was consistent with definition 7 in our study, although definition 7 failed to distinguish HPD with worse survival outcome in our cohort. In 14 patients defined as HPD by definition 3, seven patients were underestimated as non-HPD progressive disease if new lesions emerging during ICI treatment are not calculated into tumor growth. This subgroup of patients had significantly worse OS compared with non-HPD progressive disease and presented comparable OS with the rest seven patients with HPD. Collectively, our analysis indicated that taking new lesions or metastasis into consideration is important when estimating tumor growth pattern and definition criteria development of HPD.

Furthermore, definition 3 could differentiate worse PFS as well. Generally, tumor evaluation is performed every 6 to 8 weeks during the systematic treatment. Progressive disease was determined at the time of first imaging evaluation, which might result in the little difference of PFS among the patients with progressive disease. However, our criteria could diagnose HPD with shorter PFS. This means the optimized criteria system is able to screen out patients with not only rapid tumor growth but also the deterioration of symptoms or blood biochemical index, which could lead to more frequent imaging evaluation.

Furthermore, for patients with HPD, treatment strategy transition and more intense clinical care support are urgently needed for the even higher risk of death than non-HPD progressive disease. Therefore, a reliable definition of HPD should also have the ability to identify as many patients as possible with much poorer survival. In addition, the ease of calculation is also a factor that should be taken into consideration, which would possess widespread adoption and further validation in clinical practice. Taking all the above into consideration, we believed that definition 3 (tumor growth kinetics ratio of more than two fold that incorporated new lesions into calculation) would be better in gastrointestinal cancers.

Recently, David Gandara et al. proposed the definition of “fast progression,” defined as ≥50% increase in the sum of largest diameters of target lesions within 6 weeks post-treatment, or death due to disease progression within 12 weeks if post-treatment scan was infeasible. It was indicated that fast progression NSCLC patients could not benefit from ICI, as OS of fast progression patients was similar between atezolizumab and docetaxel (21). In 51 patients with progressive disease in our cohort, 25 patients were eligible for fast progression evaluation according to the criteria by David Gandara et al. and 7 patients were defined as fast progression. However, no significant difference was found in OS between patients with and without fast progression (hazard ratio 0.41, 95% CI 0.12–1.44; P = 0.1778).

As is well known, different tumor types present diverse tumor biological behavior. It should be carefully studied whether different tumor types could be evaluated with the same definition. For example, patients with neuroendocrine tumors seem to be more likely to progress quite fast, with three out of four patients with neuroendocrine tumors identified as HPD in our previous report (13). These patients might also suffer tumor progression if they receive chemo or targeted therapy. Some tumor types, such as melanoma, non-small-cell lung cancer, and urothelial cancer, have been shown to benefit from ICIs (22–24). However, clinical benefit is limited in gastric cancer treated with ICI monotherapy (5, 25). In the present study, we developed HPD criteria in gastrointestinal cancer. Further validation in other tumor types is still needed in the future.

A reliable definition is also the foundation of exploring risk factors associated with HPD, which will in turn provide the possibility of avoiding ICI treatment in patients with high risk of HPD. Several studies have investigated the factors associated with the rapid tumor progression (10, 17, 26–29). Blood neutrophil-to-lymphocyte ratio (NLR) and platelet-to-lymphocyte ratio (PLR) have been previously reported to measure the immune microenvironment and inflammatory status (30). During the treatment of ICIs, baseline and dynamic changes of NLR were shown to be related to disease progression in several cancers, such as non-small cell lung cancer, small cell lung cancer, and melanoma (31–34). However, only few studies focused on HPD risk factors of gastrointestinal cancer. A study of gastric cancer indicated that PD-1 inhibitors may promote the proliferation of the tumor infiltrating regulatory T cells, which might explain the inhibition of antitumor immunity (20). Our analysis showed that baseline and on-treatment variant of NLR and PLR were not related with occurrence of HPD. We also did additional analysis to validate if NLR and PLR were associated with tumor progression on ICI treatment in our cohort. Results indicated that baseline NLR and PLR and dynamically increased NLR during ICI therapy were associated with the tumor progression (**Supplementary Table 1**). These results indicated NLR and PLR as a worse prognostic biomarker in gastrointestinal cancer treated with ICI, but not a specific risk factor for HPD.

Some genomic mutations were indicated to be associated with occurrence of HPD. Alterations in SMARCA2 gene and APC signaling pathway might be associated with the higher risk of HPD, while patients with MSH6 gene or Wnt signaling pathway alteration might have lower risk to develop HPD. SMARCA2 gene belong to the SWI1/SNF1 family that are responsible chromatin modifying (35). Variates in the main SMARCA genes could lead to loss of expression of their respective proteins within the nucleus, further impair both CD4 silencing and CD8 activation, and might relate to inferior ICI response (36, 37), which might help explain the association between SMACAR2 mutation and higher risk of HPD. Although microsatellite status was not statistically associated with the emergence of HPD, patients with dMMR/MSI-H had numerical lower incidence of HPD (5.6% in MSI-H subgroup *vs* 17.6% in MSS subgroup). That might explain the protective effect of MSH6 mutation. The risk factors of HPD still needs further investigations.

There are several limitations in this study. Firstly, this study was conducted in a group of gastrointestinal cancer patients. Further investigations with more tumor types are warranted. Secondly, the retrospective nature and small sample size of HPD events in our study relatively limit the generalizability of our conclusion. Prospective studies with larger sample size are needed for the elucidation of the HPD occurring during immunotherapy. Thirdly, the aim of this study is to evaluate the performance of HPD under different definitions and to provide the preferable criteria for HPD evaluation; thus, patients treated with combination therapy were excluded to minimize the confounding factors in the study. As ICI combination therapy is widely adopted nowadays (38, 39), incidence and tumor growth pattern of HPD in combination therapy need further investigation.

## Conclusion

This study provided the first comprehensive comparison on the different definition systems of HPD during immunotherapy in gastrointestinal cancer. Adding new lesions emerging during the treatment was shown to be reliable for the assessment of tumor growth kinetics. Tumor growth kinetics serves as a better way to reflect tumor growth acceleration compared with bidimensional assessment with tumor diameters. Genomic alterations were indicated to be associated with the occurrence of HPD. Further studies with larger sample size and multiple tumor types are needed.

## Data Availability Statement

The original contributions presented in the study are included in the article/**Supplementary Material**. Further inquiries can be directed to the corresponding authors.

## Ethics Statement

The studies involving human participants were reviewed and approved by the Ethics Committee of Beijing Cancer Hospital (Approval ID: 2021YJZ34). The patients/participants provided their written informed consent to participate in this study.

## Author Contributions

Conception and design: ZW, CL, YB, JL, and LS. Development of methodology: ZW, YB, XCZ, and LC. Acquisition of data (provided animals, acquired and managed patients, provided facilities, etc.): ZW, CL, ZP, XTZ, XW, JL, and LS. Analysis and interpretation of data (e.g., statistical analysis, biostatistics, computational analysis): ZW, YB, XCZ, ZZ, and LC. Writing, review, and/or revision of the manuscript: ZW, XCZ, CL, ZZ, LC, and LS. Administrative, technical, or material support (i.e., reporting or organizing data, constructing databases): ZW, CL, and LS. Study supervision: JL, LS.

## Funding

This work was supported by the National Key Research and Development Program of China (No. 2017YFC1308900), the Major Program of National Natural Science Foundation of China (No. 91959205), and Beijing Hospitals Authority Clinical Medicine Development of Special Funding Support, code: (ZYLX202116).

## Conflict of Interest

XCZ, LC, and ZZ are employed by 3D Medicines Inc.

The remaining authors declare that the research was conducted in the absence of any commercial or financial relationships that could be construed as a potential conflict of interest.

## Publisher’s Note

All claims expressed in this article are solely those of the authors and do not necessarily represent those of their affiliated organizations, or those of the publisher, the editors and the reviewers. Any product that may be evaluated in this article, or claim that may be made by its manufacturer, is not guaranteed or endorsed by the publisher.

## Supplementary Material

Supplementary Figure 1 Kaplan-Meier plots of overall survival (OS) in patients with HPD compared with non-HPD progressive disease defined by **(A)** definition 1, **(B)** definition 2, **(C)** definition 4, **(D)** definition 5, **(E)** definition 6, and **(F)** definition 7. HPD, hyperprogressive disease; PD, progressive disease; HR, hazard ratio; CI, confidence interval.

Supplementary Figure 2 Kaplan-Meier plot of overall survival (OS) between subgroups of discordant HPD, concordant HPD, and non-HPD progressive disease when including new lesions into the calculation of tumor growth or not. HPD, hyperprogressive disease; PD, progressive disease.

Supplementary Figure 3 Genomic alterations from NGS analysis with ctDNA derived from blood samples collected prior ICI treatment. NGS, next-generation sequencing; ctDNA, circulating tumor DNA; ICI, immune checkpoint inhibitors; HPD, hyperprogressive disease; PD, progressive disease; dMMR, mismatch repair deficient; MSI-H, microsatellite instability-high.
