# Supplementary figures and images for: Redefine Hyperprogressive Disease During Treatment With Immune-Checkpoint Inhibitors in Patients With Gastrointestinal Cancer
Source: Front Oncol. 2021 Nov 9;11:761110. doi: 10.3389/fonc.2021.761110 (PMC8630635; doi:10.3389/fonc.2021.761110)

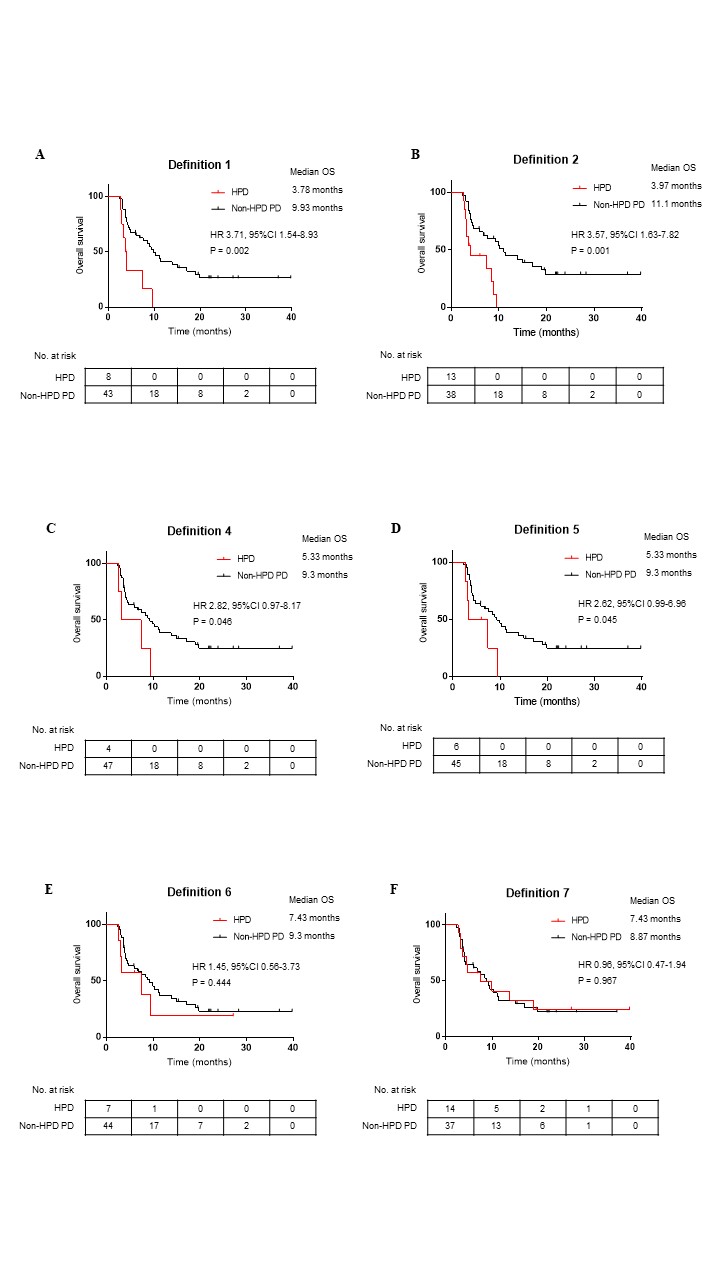

Supplement: Supplementary Figure 1 — Kaplan-Meier plots of overall survival (OS) in patients with HPD compared with non-HPD progressive disease defined by (A) definition 1, (B) definition 2, (C) definition 4, (D) definition 5, (E) definition 6, and (F) definition 7. HPD, hyperprogressive disease; PD, progressive disease; HR, hazard ratio; CI, confidence interval. [file Image_1.jpeg]

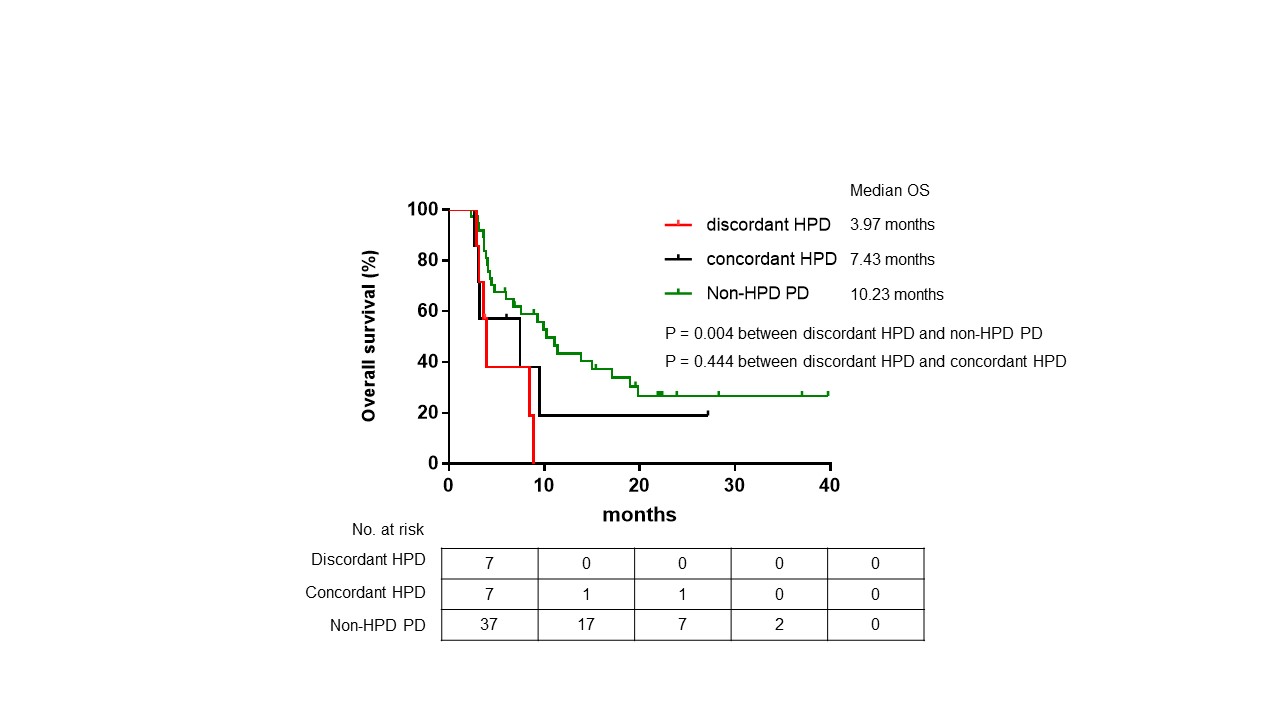

Supplement: Supplementary Figure 2 — Kaplan-Meier plot of overall survival (OS) between subgroups of discordant HPD, concordant HPD, and non-HPD progressive disease when including new lesions into the calculation of tumor growth or not. HPD, hyperprogressive disease; PD, progressive disease. [file Image_2.jpeg]

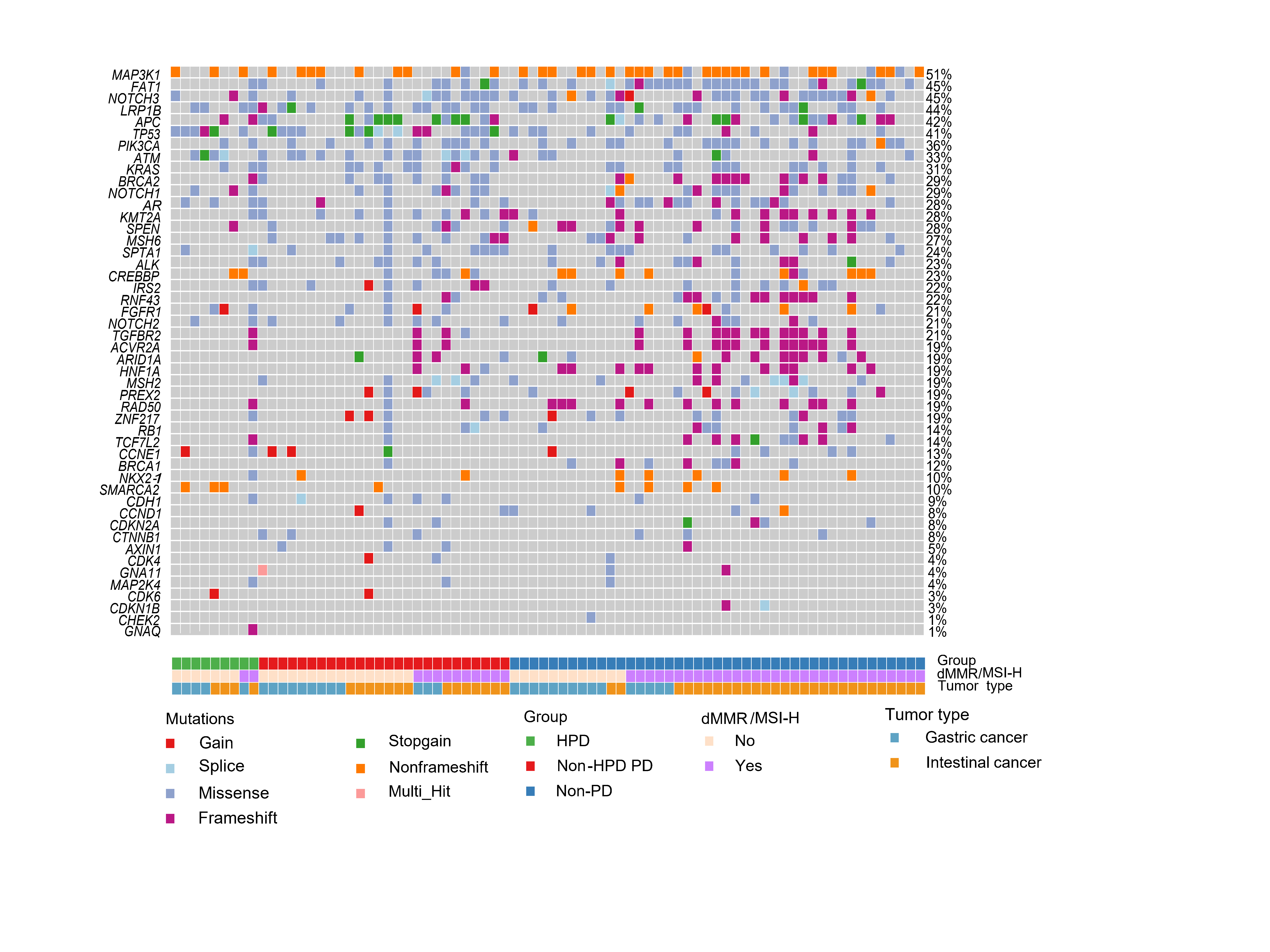

Supplement: Supplementary Figure 3 — Genomic alterations from NGS analysis with ctDNA derived from blood samples collected prior ICI treatment. NGS, next-generation sequencing; ctDNA, circulating tumor DNA; ICI, immune checkpoint inhibitors; HPD, hyperprogressive disease; PD, progressive disease; dMMR, mismatch repair deficient; MSI-H, microsatellite instability-high. [file Image_3.jpeg]
